# Retraction: Cross border project in China-Pakistan economic corridor and its influence on women empowerment perspectives

**DOI:** 10.1371/journal.pone.0314377

**Published:** 2024-11-20

**Authors:** The PLOS ONE Editors

Following the publication of this article [1] and Correction [2], multiple concerns were raised.

The article [1] reports conclusions about the direct economic benefits of CPEC that are not supported by the methodology and results. A member of the *PLOS ONE* Editorial Board agreed with this concern about the reliability of these conclusions.

The article [1] does not provide sufficient information to clarify how participants were recruited, and conflicting information was provided in post-publication discussions. The *PLOS ONE* Editors remain concerned about whether the recruitment process was adequate to ensure recruitment of an appropriate and independent sample population.

Ethics approval documents provided in post-publication discussions were not sufficient to confirm compliance with the PLOS Human Subjects Research policy.

Inaccuracies in reporting include:

- In the underlying dataset (S1 File in [2]), several of the average values in the EOAVE, EIAVE, CPECAVE, and QOLAVE columns don’t match the calculated mean of the data in the corresponding columns. The corresponding author acknowledged that errors occurred during data processing.
- The article [1] states that interviews were performed with women, however three of the four interviews were with men. These interviews were also reported in an earlier article from the same author group [3], and there are discrepancies between the interview quotes in the two articles [1, 3] and transcripts provided to PLOS in post-publication discussions. The corresponding author stated that transcription of the interviews involved paraphrasing, and that some quotes were reattributed to fit the narrative better.
- In the article [1], references 16 and 57 are cited inappropriately to support claims about the benefits of CPEC in Pakistan. The corresponding author confirmed that these were cited in error.

The *PLOS ONE* Editors retract this article due to concerns about the reliability and validity of the conclusions, accuracy of reporting, and compliance with the PLOS Human Subjects Research policy.

JKumar and JKumari did not agree with the retraction and stand by the article’s findings. JKumar apologizes for the issues with the published article. CX and MI either did not respond directly or could not be reached.
